# The Validity and Reliability of the Copenhagen Burnout Inventory for Examination of Burnout among Preschool Teachers in Serbia

**DOI:** 10.3390/ijerph18136805

**Published:** 2021-06-24

**Authors:** Pavle Piperac, Jovana Todorovic, Zorica Terzic-Supic, Aleksandra Maksimovic, Svetlana Karic, Filip Pilipovic, Ivan Soldatovic

**Affiliations:** 1 Department of Humanities, Faculty of Medicine, University of Belgrade, Pasterova 2, 11000 Belgrade, Serbia; 2 Faculty of Medicine, Institute of Social Medicine, University of Belgrade, Dr Subotica 15, 11000 Belgrade, Serbia; jovana.todorovic@med.bg.ac.rs (J.T.); zorica.terzic-supic@med.bg.ac.rs (Z.T.-S.); 3 Faculty of Science, University of Kragujevac, Radoja Domanovica 12, 34000 Kragujevac, Serbia; aleksandra.maksimovic@pmf.kg.ac.rs; 4 Department of Studies for Preschool and Nursery Teachers, Academy of Professional Studies, Dobropoljska 5, 15000 Sabac, Serbia; svetlana.karic@gmail.com; 5 Institute for Orthopedics Banjica, Mihaila Avramovica 28, 11000 Belgrade, Serbia; drfilippilipovic@gmail.com; 6 Faculty of Medicine, Institute for Medical Statistics and Informatics, University of Belgrade, Dr Subotica 15, 11000 Belgrade, Serbia; ivan.soldatovic@med.bg.ac.rs

**Keywords:** burnout, preschool teachers, Copenhagen Burnout Inventory, validation

## Abstract

Introduction: Burnout syndrome is being increasingly recognized as a factor that affects the health status and is being examined among different professional groups. Consequently, there is a need for a reliable and valid instrument for its examination. Teachers are emerging as a professional group of interest in the area of burnout research, so the aim of this study was to assess the validity and reliability of the Serbian version of Copenhagen burnout inventory among teachers at preschool institutions in Serbia. Materials and Methods: This research was conducted as a cross-sectional study between October 2018 and April 2019 on a nationally representative sample of preschool teachers in Serbia. The internal consistency of the scale was assessed using Cronbach’s alpha, and the construct validity was examined using exploratory (EFA) and confirmatory factor analyses (CFA). Results: The average score on total burnout was 39.1 ± 17.0, while the average score per scales on the Copenhagen burnout inventory was: 41.3 ± 18.7 for personal burnout, 41.2 ± 15.9 for work-related burnout, and 34.7 ± 22.0 for client-related burnout. The Cronbach’s alpha for the entire scale was 0.936, the Cronbach’s alpha for the personal burnout scale was 0.906, and the Cronbach’s alpha for the work-related burnout scale was 0.765, while the Cronbach’s alpha for the client-related burnout scale was 0.901. The EFA for the CBI showed three factors. The factor loadings varied from 0.575 to 0.859. The three factors explained 67.17% of the variance. Conclusions: Our study showed that the three-factor Serbian version of the Copenhagen burnout inventory can be used for the assessment of burnout syndrome among teachers.

## 1. Introduction

Burnout is defined as a “syndrome conceptualized as resulting from chronic workplace stress that has not been successfully managed” in the International Classification of Diseases 11th revision (ICD-11) [1]. It is characterized by a feeling of insufficient energy or exhaustion, increased mental distance from one's work, or a feeling of negativity or cynicism about one's work and reduced professional efficiency [1]. The development of burnout syndrome (burnout) can be manifested through psychological and somatic symptoms, such as constant irritability, anxiety, insomnia, forgetfulness, problems with concentration, depression, arrhythmia, hypertension, gastrointestinal problems, and headaches [2,3,4,5]. As these symptoms are unspecific, burnout is usually unrecognized at the early stages [5,6,7].

The first signs of burnout may be chronic work-related stress, mental or physical exhaustion, and strain [3,8,9]. These symptoms can be accompanied by behavioral problems through intentional later arrivals at work, the postponement of obligations and reluctance to accept new obligations, and/or prolonging work breaks [6,10]. Workers with burnout report a loss of interest in the job, distancing from the job, and not understanding the job as meaningful [6,10]. There could also be a sense of diminished self-confidence and personal achievement [11], decreased self-efficacy [12], and income dissatisfaction [13]. Early detection of burnout can help improve one’s well-being and general mental health [7,14]. 

Workplace stress is a frequent and well-established problem among teachers at various educational levels, and studies in different regions of the world have shown that stress associated with the work in educational settings is significant [7,13,15,16] and that almost 70% of teachers at some point during their working life show symptoms of burnout [7]. 

The job of a teacher in a professional sense is very responsible and demanding. Teacher burnout can severely disrupt professional goals and productivity in the preschool environment, resulting in serious negative consequences for the work, teachers' health, and teacher–child interactions [13]. In addition to children, preschool teachers communicate almost daily with children's parents, who can be demanding, and at the same time, they should maintain good relations and cooperation with colleagues and superiors [6]. During the sensitive developmental period of the preschool age, children spend a lot of time learning through interactions with their teachers in preschool institutions [17]. In addition to regular preschool programs, teachers are often involved in advanced activities and special programs for working with children [6]. Burnout among preschool teachers has received increasing attention among researchers, because the early influence of a preschool educator’s relationship and communication can guide future school success and a child’s social and emotional development [17]. As other professionals providing social services, preschool teachers are shown to be at risk for developing burnout syndrome [18]. It is considered that the work of preschool teachers is more complex than the work of teachers at other educational levels, as their daily tasks are of greater number, more challenging, and include more contact with parents [19]. Additionally, in numerous countries, preschool teachers have lower salaries and often longer working hours compared to the teachers at other educational levels [18]. A study among the preschool teachers in Greece showed high levels of emotional exhaustion among them [20], while the study of preschool teachers in Turin, Italy showed that around a half of preschool teachers report medium or high levels of emotional exhaustion or depersonalization [18]. Some instruments have been specifically developed for the examination of burnout among the teacher population, such as the Teacher of Physical Education Burnout Inventory [21], the Teachers Burnout Scale [22], Maslach Burnout Inventory—Educators Survey [23], and instruments that could be applied in different professional settings [24,25,26,27,28,29,30,31,32]. The most commonly used instrument is the Maslach Burnout Inventory (MBI) [20], including its specific versions and subscales. MBI is not widely available for researchers because of its commercial-only use. There is also a lack of adaptability to different cultural specificities, and as it is developed for use among human service professionals—among professionals who work with people—it cannot be implemented in other occupations [24,33]. Many authors developed instruments available for free use with the aim to overcome these challenges. The Copenhagen Burnout Inventory (CBI) has been used in many studies since its development, and it has been proven to be a valid and reliable instrument [24,33]. The CBI has been translated into many languages and adapted in different countries [14,24,33,34,35,36,37,38,39,40,41,42,43]. One of the main advantages of the Copenhagen burnout inventory is that it examines burnouts in three independent dimensions: personal burnout, work-related burnout, and client-related burnout and measures the total burnout for the three scales combined. Additionally, the first part of the instrument, the personal burnout scale, can be used in variety of populations independently of a person’s employment status [33].

In Serbia, burnout was examined among recruiters in human resource services of different companies [44], students of the University of Belgrade [45,46,47], special education professionals [48,49], social workers [50], pharmacists [51], professionals in the pharmaceutical manufacturing industry and marketing [52,53], underground coal miners [54], formal caregivers of children with cerebral palsy [55], workers in the food industry [56], and the most common among healthcare professionals [57,58,59,60]. The majority of these studies examined burnout using the Maslach Burnout Inventory. 

The research interest in burnout is increasing, and although many studies have been conducted so far in Serbia, this rising interest leads to a need for a reliable and valid instrument that could be easily applied in different population groups. The aim of this study was to assess the validity and reliability of the Serbian version of the Copenhagen burnout inventory (Supplementary Materials File S1) among teachers at preschool institutions in Serbia. 

## 2. Materials and Methods

This research was conducted as a cross-sectional study between October 2018 and April 2019 on a nationally representative sample of preschool teachers in Serbia. The study included a total of 475 participants.

### 2.1. Research Instrument

Copenhagen Burnout Inventory (CBI) [24] is a 19-item scale measuring burnout in three domains: personal burnout (PB) (6 items: questions 1–6), work-related burnout (WRB) (7 items: questions 7–13), and client-related burnout (CRB) (6 items: questions 14–19). The questionnaire was specifically adapted for the purposes of our research, and the domain on CRB was adapted to children-related burnout. The answers for the CBI were given on a five-point Likert scale, ranging from 0 to 4, where 0 means never and 4 means always. The answers were then transformed into percentages of time: 0 = 0%, 1 = 25%, 2 = 50%, 3 = 75%, and 4 = 100%, as per the instructions given by the authors of the questionnaire [24]. The score on each scale was calculated as the average percentage of the score of the questions of that scale, and the total score was calculated as the average score of the three scales together. The CBI was translated to the Serbian language and back, according to the recommendations by the World Health Organization [61]. A pilot testing was conducted on 20 participants to examine whether the questions in the questionnaire were clear, understandable, whether their order was correct, and to determine the time required to complete the questionnaire. These 20 participants filled in the questionnaire once more 10 days later to allow the calculation of the intraclass correlation coefficient (ICC).

We analyzed a total of four variables: score on Copenhagen burnout inventory, score on personal burnout scale, score on work-related burnout scale, and score on client-related burnout scale. 

### 2.2. Data Collection Procedure

The sample size for the precision of 5% was calculated to be 401. The sampling frame included the data from the Republic Institute of statistics on the number of preschool teachers in Serbia. The sample was drawn from the statistical regions of Serbia (Belgrade, Northern Serbia, Central and Western Serbia, and Eastern and Southern Serbia), and each region represented a different cluster. The sample size for each cluster was calculated proportionally. The preschool institutions, which were included in the study, were then randomly selected for each cluster. Preschool teachers who were at the workplace during the morning shift on the day of the research were included in this study. All respondents were given information about the processes and aims of the research, and then, they were asked to fill out an anonymous questionnaire. We considered that all respondents who completed and returned the questionnaires gave their consent to participate in the research. This study was approved by the ethical committee of the Faculty of Medicine, University of Belgrade (No.1550/XI-49). 

### 2.3. Statistical Analyses

The methods of descriptive and analytical statistics were applied. The internal consistency of the scale was assessed using Cronbach’s alpha. The test–retest consistency was assessed using the intraclass correlations coefficients (ICC). For the factor analysis, the total sample was randomly divided into two samples, each including approximately 50% of the participants. 

The exploratory factor analysis (EFA) was done to explore the factor structure and was done on one-half of the sample. The extraction of factors was done using Promax rotation, as the hypothesis was that the factors were correlated. Values for the Kaiser–Meyer–Olkin (KMO) measure of sampling adequacy and Bartlett’s test of sphericity (preferably significant) were used to assess the suitability of the data for factorization. The criterion for loading and cross-loading was set at 0.4. The confirmatory factor analysis (CFA) was done on the second half of the sample (selected randomly). The goodness of fit indices used were the goodness of fit index (GFI), adjusted goodness of fit index (AGFI), the comparative fit index (CFI), and the root mean error of approximation (RMSEA). The CFA was done using SPSS Amos 22.0 (Windows, Armonk, NY, USA). The missing values were replaced using the series mean. The analyses were done using the Statistical Package for Social Sciences (SPSS) for Windows 22.0 (Armonk, NY, USA).

## 3. Results

The study included a total of 475 preschool teachers in Serbia. The majority of our participants were female (458/475—96.4%), with an average age of 39.5 ± 8.2 years, and more than four-fifths of them (85.2%) lived in urban areas. More than two-thirds of the participants were married (67.5%) and had children (72.6%). Around three-quarters of the participants (75.2%) had tenured contracts, and the average length of work experience was 14.5 ± 7.8 years. Just under a half of our participants (43.3%) worked with children aged three to four years, 27.6% of them worked with children of five to six years of age, and 18.6% of participants worked with children at the age of six. The rest of our participants could not state the ages of the children they work with, as the groups were mixed, or they changed the groups frequently. 

The average score on total burnout was 39.1 ± 17.0, while the average score per scales of the CBI was: 41.3 ± 18.7 for PB, 41.2 ± 15.9 for WRB, and 34.7 ± 22.0 for CRB.

The Cronbach’s alpha for the entire scale was 0.936, the Cronbach’s alpha for the PB scale was 0.906, and the Cronbach’s alpha for the WRB scale was 0.765, while the Cronbach’s alpha for the CRB scale was 0.901. The test–retest reliability was: ICC = 0.754. 

The average scores and reliability of the scales are presented in Table 1.

The highest average score per item of the CBI was for item 7—WRB1 (“Work emotionally exhausting”) and the lowest for item 15—CRB2 (“Frustrating to work with children”). No item indicated problematic amounts of Skewness or Kurtosis. Their values revealed acceptable levels; therefore, no transformations were used to correct the non-normality. The average scores per item of the CBI are presented in Table 2. 

The EFA for the CBI showed three factors. The factor loadings varied from 0.575 to 0.859. The three factors explained 67.17% of the variance: factor 1—53.78%, factor 2—7.59%, and factor 3—5.80%. The Kaiser–Meyer–Olkin measure of the sampling adequacy was 0.943, and Bartlett’s test of sphericity was statistically significant (*p* < 0.001). 

The CFA of the three-factor model of the CBI showed that the GFI was 0.781, the AGFI was 0.720, the CFI was 0.869, and the RMSEA was 0.113 (95% CI: 0.104-0.122). 

The factors and factor loadings for each item using the principal component analysis with Promax rotation and Kaiser normalization—CBI are presented in Table 3. 

The correlation matrix between the components is presented in Table 4.

## 4. Discussion

Our study examined the validity and reliability of the Serbian version of the CBI among teachers who work in preschool institutions in Serbia. Our study showed that the average scores on the PB, WRB, and CRB scales were higher than those demonstrated previously in studies examining burnout among preschool teachers [24,62]. Our results were more similar to the findings in the samples of secondary school teachers [14]. 

We showed that the CBI has good reliability and validity and could be used for the assessment of burnout among preschool teachers. The CBI showed high internal consistency, and the Cronbach’s alphas varied from 0.765 to 0.936, which were similar to those previously reported [40,41,63,64]. There were low floor and ceiling effects (0.2–7.2%) in our study, as previously reported [63]. The EFA showed three factors with good factor loadings and explained almost 70% of the variance. The factors partially corresponded to the scales in the instrument. In our study, all of the items in the PB scale were in the same factor (factor 1), as were all of the items regarding CRB (factor 2). However, the items regarding WRB in the original scale were, in our EFA, distributed in all three of the factors. The four items (out of seven) were in one factor (factor 3), one was in factor 1, along with the items regarding PB, and the other items were in factor 2 with the items regarding CRB. The items in the domain of PB refer to the personal exhaustion, and the items in the domain of WRB reflect only the work-related exhaustion experienced by the participants [24]. Both PB and WRB assess the degree of exhaustion of the individual. Many workers today spend most of their time at the workplace and, therefore, work schedules including shift work can disturb participants’ perception of separating personal and work-related exhaustion. The strong correlations between these factors were reported in previous studies [39,62]. This can probably explain in our study why item 10—WRB4 (”Feel worn out at the end of the day”) was in factor 1, along with the items regarding PB.

Previous studies have shown that the WRB scale has the lowest factor loadings [41,63], which was partially true for our study, as the factor loadings varied between 0.575 and 0.859, and this factor included the items with the lowest and the highest loadings. A recent study from Serbia examined the psychometric properties of the WRB scale, and their findings indicated that the WRB scale could be particularly useful as a short, two-dimensional scale for measuring two particular aspects of burnout, work frustration and work exhaustion, in different occupations [65]. The items from the WRB scale were in all three of the factors in our factor analysis, and the factor distribution did not correspond to the ones in the previously mentioned study [65]. 

Item 13—WRB7 (“Time for friends and family”) was, in previous studies, shown to have low factor loadings [33,39,43], and this may be related to the inverse scoring of the item. This item is the only one in the questionnaire with a positive formulation, and all the others have a negative response direction, so it is possible that participants continued with the response stereotype [24,62]. This item was also the item with the lowest factor loading in our study.

Our findings are significant, as the problem of burnout is being increasingly examined among different populations and is now not limited only to human service professionals. The problem of burnout among teachers and, especially, of teachers of young children is beginning to receive more attention [12,13,16]. The teachers of preschool children significantly influence their development, and their poor job performance can have a negative influence not only on them as individuals but on the children they are trusted with [6,12].

One of the possible limitations of our study is the lack of data regarding the association of burnout with communication with parents as the possible clients of the preschool teachers, along with the children. In our study, children are clearly defined as clients, but we have no information about burnout being associated with the parents. The authors of the original questionnaire proposed to replace the term “clients” in the questionnaire with the appropriate term depending on the population examined. In many professions, the clients may refer to more than one group. Students communicate with their colleagues and faculty members, but pediatricians communicate with children and their parents, as do social workers and teachers. A thorough examination of the association of burnout among teachers with the communication with parents can bring new and valuable insights to the concept of burnout in this population. 

The main strength of our study was in its sample, as the study was conducted on a nationally representative sample of preschool teachers and included teachers from urban and rural environments. The inclusion of a diverse sample of preschool teachers enabled the generalizability of our results.

## 5. Conclusions

Our study showed that the Serbian version of the CBI can be used for the assessment of burnout among teachers who work in preschool institutions, as the instrument has a good test–retest reliability, good internal validity, and acceptable construct validity. However, further analyses of the scale on larger samples and on samples of teachers at various educational levels should be conducted to adequately examine the scale. Burnout is still not well-examined among teachers, especially among preschool teachers, and as we can expect the number of studies on this population to increase, it is valuable to have a good instrument that is also convenient to use. 

## Figures and Tables

**Table 1:** Mean scores on the CBI scale and Cronbach’s alpha.

Scale	Average Score X ± SD	Cronbach’s alpha	Floor *n* (%)	Ceiling *n* (%)
Total burnout CBI	39.1 ± 17.0	0.936	2 (0.4)	0 (0)
Personal burnout CBI	41.3 ± 18.7	0.906	10 (2.1)	1 (0.2)
Work-related burnout CBI	41.2 ± 15.9	0.765	6 (1.3)	0 (0)
Client-related burnout CBI	34.7 ± 22.0	0.901	34 (7.2)	1 (0.2)

CBI—Copenhagen Burnout Inventory.

**Table 2:** Average scores per item of the CBI.

Item	Score	SD	Skewness	Kurtosis
PB1	50.87	21.52	−0.150	−0.283
PB2	48.47	22.26	−0.193	−0.293
PB3	41.28	23.95	0.132	−0.601
PB4	34.69	25.40	0.257	−0.833
PB5	39.38	24.56	0.059	−0.763
PB6	37.53	22.25	0.258	−0.395
WRB1	51.52	26.39	−0.268	−0.376
WRB2	44.55	24.17	−0.091	−0.422
WRB3	28.70	23.01	0.310	−0.672
WRB4	47.17	26.23	0.125	−0.505
WRB5	28.92	24.19	0.437	−0.537
WRB6	26.63	22.95	0.550	−0.282
WRB7	34.22	27.62	0.396	−0.716
CRB1	42.76	27.65	0.048	−0.556
CRB2	26.20	25.19	0.547	−0.644
CRB3	41.50	27.10	0.082	−0.744
CRB4	38.40	27.10	0.320	−0.556
CRB5	31.48	25.38	0.343	−0.629
CRB6	34.91	30.05	0.412	−0.845

PB — Personal Burnout; WRB — Work Related Burnout; CRB — Client Related Burnout.

**Table 3:** Factors and factor loadings for each item using the principal component analysis with Promax rotation and Kaiser normalization—CBI.

Item	Item Description	Factor 1	Factor 2	Factor 3
PB1	Feel tired	0.845		
PB2	Physically exhausted	0.841		
PB5	Feel worn out	0.824		
PB4	Cannot take it anymore	0.815		
PB3	Emotionally exhausted	0.781		
WRB4	Feel worn out at the end of the day	0.744		
PB6	Feel weak and susceptible to illness	0.718		
CRB3	Working with children is exhausting		0.854	
CRB1	Find it hard to work with children		0.842	
CRB6	Able to continue working with children		0.800	
CRB4	Give more than get back		0.776	
WRB2	Work exhausting		0.765	
CRB5	Tired working with children		0.757	
CRB2	Frustrating to work with children		0.755	
WRB1	Work emotionally exhausting		0.703	
WRB6	Feel that every working hour is tiring			0.859
WRB5	Feel exhausted in the morning			0.856
WRB3	Work frustrates			0.793
WRB7	Time for friends and family *			0.575

* Inverse scoring item; PB — Personal Burnout; WRB — Work Related Burnout; CRB — Client Related Burnout.

**Table 4:** Component correlation matrix between the principal component analysis and Promax rotation with Kaiser normalization.

Component	Factor 1	Factor 2	Factor 3
Factor 1	1.000	0.611	0.584
Factor 2	0.611	1.000	0.605
Factor 3	0.584	0.605	1.000

## Data Availability

Data will be made available upon a request.

## Supplementary Materials

The following is available online at https://www.mdpi.com/article/10.3390/ijerph18136805/s1, Supplementary File S1.
