# Supplementary material for: The Validity and Reliability of the Copenhagen Burnout Inventory for Examination of Burnout among Preschool Teachers in Serbia
Source: Int J Environ Res Public Health. 2021 Jun 24;18(13):6805. doi: 10.3390/ijerph18136805 (PMC8297089; doi:10.3390/ijerph18136805)
Supplement: Supplementary file 1 [file ijerph-18-06805-s001.zip › ijerph-1244592-supplementary.pdf]

Kopenhagen upitnik o sagorevanju na poslu- Srpska verzija

I CBI – Lični faktori sagorevanja

U ovom delu upitniku nalazi se skup pitanja. Pažljivo pročitajte svako od njih. Zatim odaberite odgovor koji najbolje opisuje kako se osećate, prateći učestalost:

□ Nikad (0% vremena) □ Retko (25% vremena) □ Ponekad (50% vremena) □ Često (75% vremena)

□ Uvek (100% vremena)

1. **Koliko često se osećate umorno?**
   □ Nikad □ Retko □ Ponekad □ Često □ Uvek
2. **Koliko često se osećate fizički iscpljeno?**
   □ Nikad □ Retko □ Ponekad □ Često □ Uvek
3. **Koliko često se osećate emocionalno iscpljeno?**
   □ Nikad □ Retko □ Ponekad □ Često □ Uvek
4. **Koliko često pomislite „Ne mogu više ovako“?**
   □ Nikad □ Retko □ Ponekad □ Često □ Uvek
5. **Koliko često se osećate istrošeno?**
   □ Nikad □ Retko □ Ponekad □ Često □ Uvek
6. **Koliko često se osećate slabo i podložno bolestima?**
   □ Nikad □ Retko □ Ponekad □ Često □ Uvek

II CBI – Sagorevanje povezano sa poslom

7. **Da li je Vaš posao emocionalno iscrpljući?**
   □ Nikad □ Retko □ Ponekad □ Često □ Uvek
8. **Da li se osećate iscrpljeno zbog svog posla?**
   □ Nikad □ Retko □ Ponekad □ Često □ Uvek
9. **Da li Vas Vaš posao frustrira?**
   □ Nikad □ Retko □ Ponekad □ Često □ Uvek
10. **Da li se osećate istrošeno na kraju radnog dana?**
    □ Nikad □ Retko □ Ponekad □ Često □ Uvek
11. **Da li se osećate iscpljeno ujutru pri pomisli na još jedan dan na poslu?**
    □ Nikad □ Retko □ Ponekad □ Često □ Uvek
12. **Da li osećate da Vam je svaki radni sat naporan?**
    □ Nikad □ Retko □ Ponekad □ Često □ Uvek
13. **Da li imate dovoljno vremena za porodicu i prijatelje nakon posla?**
    □ Nikad □ Retko □ Ponekad □ Često □ Uvek

<u>III CBI – Sagorevanje povezano sa decom</u>

14. **Da li smatrate da je teško raditi sa decom?**

    □ Nikad □ Retko □ Ponekad □ Često □ Uvek

15. **Da li smatrate da je rad sa decom frustrirajući?**

    □ Nikad □ Retko □ Ponekad □ Često □ Uvek

16. **Da li rad sa decom crpi Vašu energiju?**

    □ Nikad □ Retko □ Ponekad □ Često □ Uvek

17. **Da li se osećate da više dajete nego što dobijate u radu sa decom?**

    □ Nikad □ Retko □ Ponekad □ Često □ Uvek

18. **Da li ste umorni od rada sa decom?**

    □ Nikad □ Retko □ Ponekad □ Često □ Uvek

19. **Da li se nekada pitate koliko ćete još biti u stanju da radite sa decom?**

    □ Nikad □ Retko □ Ponekad □ Često □ Uvek
